# Gingival metastasis from primary hepatocellular carcinoma: a case report and literature review of 30 cases

**DOI:** 10.1186/s12885-019-6020-7

**Published:** 2019-09-14

**Authors:** Yating Hou, Weiping Deng, Gang Deng, Linhui Hu, Chao Liu, Lishu Xu

**Affiliations:** 1 Shantou University Medical College, 22 Xinling Road, Shantou, 515040 Guangdong China; 2 Department of Gastroenterology, Guangdong Geriatrics Institute, Guangdong Provincial People’s Hospital, Guangdong Academy of Medical Sciences, 106 ZhongshanEr Road, Guangzhou, 510080 Guangdong China; 3 Department of Critical Care Medicine, The People’s Hospital of Gaozhou, 89 Xiguan Road, Gaozhou, 525200 Guangdong China; 4 Department of Pathology and Laboratory Medicine, Guangdong General Hospital, Guangdong Academy of Medical Sciences, Guangzhou, 510080 Guangdong China

**Keywords:** Gingival metastasis, Hepatocellular carcinoma, Diagnosis, Case report, Literature review

## Abstract

**Background:**

Gingival metastasis from primary hepatocellular cancer (HCC) is rare, highly malignant, and generally has no distinct symptoms. Not performing a biopsy can lead to misdiagnosis. This article reports an 87-year-old male with gingival metastasis from HCC. To gain a better insight into this disease, we also conducted a literature review of 30 cases and discussed the clinical and pathological characteristics, diagnosis, treatment and prognosis of this unusual form of liver cancer.

**Case presentation:**

An 87-year-old man was hospitalized with a chief complaint of chronic constipation and diffuse lower extremity edema. His past medical history included a three-year hepatitis B infection and a cerebral infarction 17 years prior. Imaging examination detected a massive hepatocellular carcinoma in the right liver lobe and multiple metastases in the lungs. Oral examinations revealed a reddish, cherry-sized exophytic mass on the right upper gum. The mass was tentatively diagnosed as a primary gingival tumor and was ultimately confirmed by biopsy as a metastatic carcinoma originating in the liver. The patient decided, with his guardians, to receive palliative care and not to remove the mass. Unfortunately, the patient accidentally bit the mass open; profuse bleeding ensued and local pressure exerted a poor hemostatic effect. The patient’s condition worsened, and he eventually died of multiple organ failure. We also performed a literature review and discussed 30 cases of gingival metastases from HCC. The findings indicated that these lesions affected males more than females, with a ratio of 6:1, and infiltrated the upper gingivae (63.1%) more than the lower gingivae (36.7%). Survival analysis indicated that the overall survival for patients with upper gingival metastasis was worse than for those with lower gingival metastasis, and patients receiving treatments for primary liver cancer or metastatic gingival tumors had better overall or truncated survival times.

**Conclusion:**

Gingival metastasis from primary hepatocellular carcinoma is rare, and its diagnosis has presented challenges to clinicians. To avoid a potential misdiagnosis, a biopsy is mandatory regardless of whether a primary cancer is located. Early diagnosis and treatment for primary liver cancer or metastatic gingival lesions may improve survival expectations.

## Background

Hepatocellular carcinoma (HCC) is prevalent worldwide, especially among the populations in East Asian countries [1]. Distant metastasis sites include the lungs, lymph nodes, bones, brain and gingivae [2]. Gingival metastasis from HCC has an especially high malignancy and poor prognosis, although it is traditionally regarded as a rare disease [3]. To the best of our knowledge, no more than 12 cases of gingival metastasis from HCC have been included in major literature sources, such as PubMed and Web of Science [4–16]. Nevertheless, these resources have not covered some of the relevant cases published in either English or non-English journals [3, 17–33]. In this manuscript, we reported a male patient aged 87 with gingival metastasis from HCC. Additionally, we performed a literature review of 30 cases to further discuss the clinical and pathological characteristics, diagnosis, treatments, and prognosis of gingival metastasis from HCC. This case series includes the present case and additional cases retrieved from journals published in East Asia, which has the world’s largest HCC population [1].

## Case presentation

**Fig. 1:**
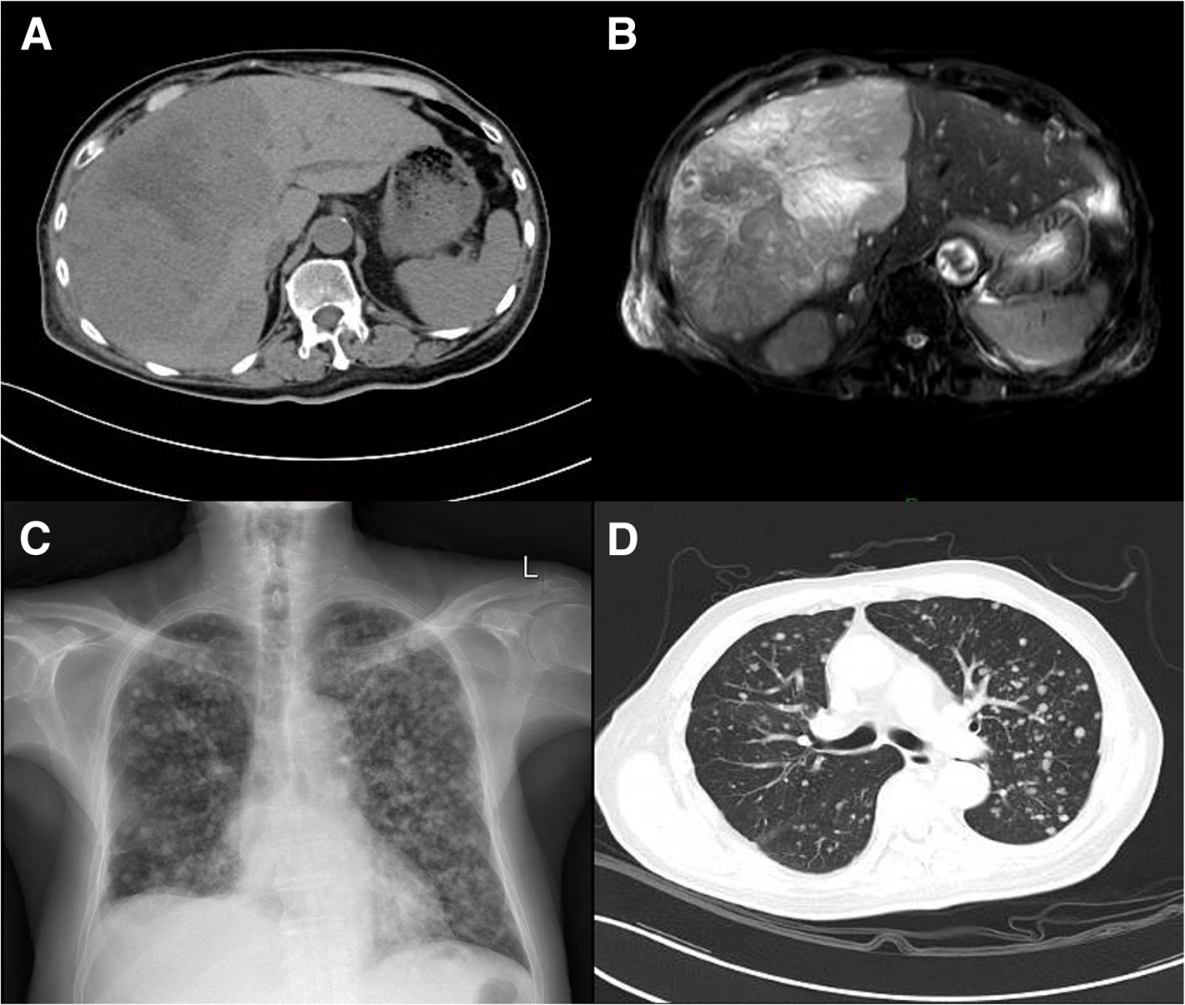
Radiographic images of the involved organs. **a** CT and **b** MR image of the primary liver mass. **c** X-ray and **d** CT image of multiple metastases to both lungs

An 87-year-old male patient with a chief complaint of chronic constipation and diffuse lower extremity edema was referred to the gastroenterology department at Guangdong Provincial People’s Hospital. A review of the patient’s past medical history revealed chronic hepatitis B infection and liver cirrhosis for 3 years, as well as depressive-anxiety neurosis and sequelae of a cerebral infarction 70 years prior. Abdominal computerized tomography (CT) and magnetic resonance imaging (MRI) scans revealed a well-defined low-density solid mass measuring approximately 15.0 × 13.0 cm in the right liver lobe surrounded by multiple nodules (Fig. 1a, b). Chest X-rays and CT scans detected multiple nodules in both lungs (Fig. 1c, d). The patient was clinically diagnosed with advanced primary liver cancer and multiple intrahepatic and lung metastases. Laboratory tests revealed anemia (hemoglobin 83 g/L), hypoproteinemia (albumin 27.7 g/L), hyponatremia (Na^+^ 125.8 mmol/L), and hyperammonemia (ammonia 65.0 µmol/L). Elevated serum levels of creatine (Cr, 105.1 µmol/L), total bilirubin (TBIL, 25.3 µmol/L), and gamma-glutamyl transpeptidase (GGT, 379 U/L), as well as impaired blood clotting function [International normalized ratio (INR), 1.22; activated partial thromboplastin time (APTT), 46.8 s] were reported. A significantly elevated level of carbohydrate antigen-125 (CA-125, 163.8 U/L) was also disclosed; however, the serum level of alpha-fetoprotein (AFP) was within the normal range.

**Fig. 2:**
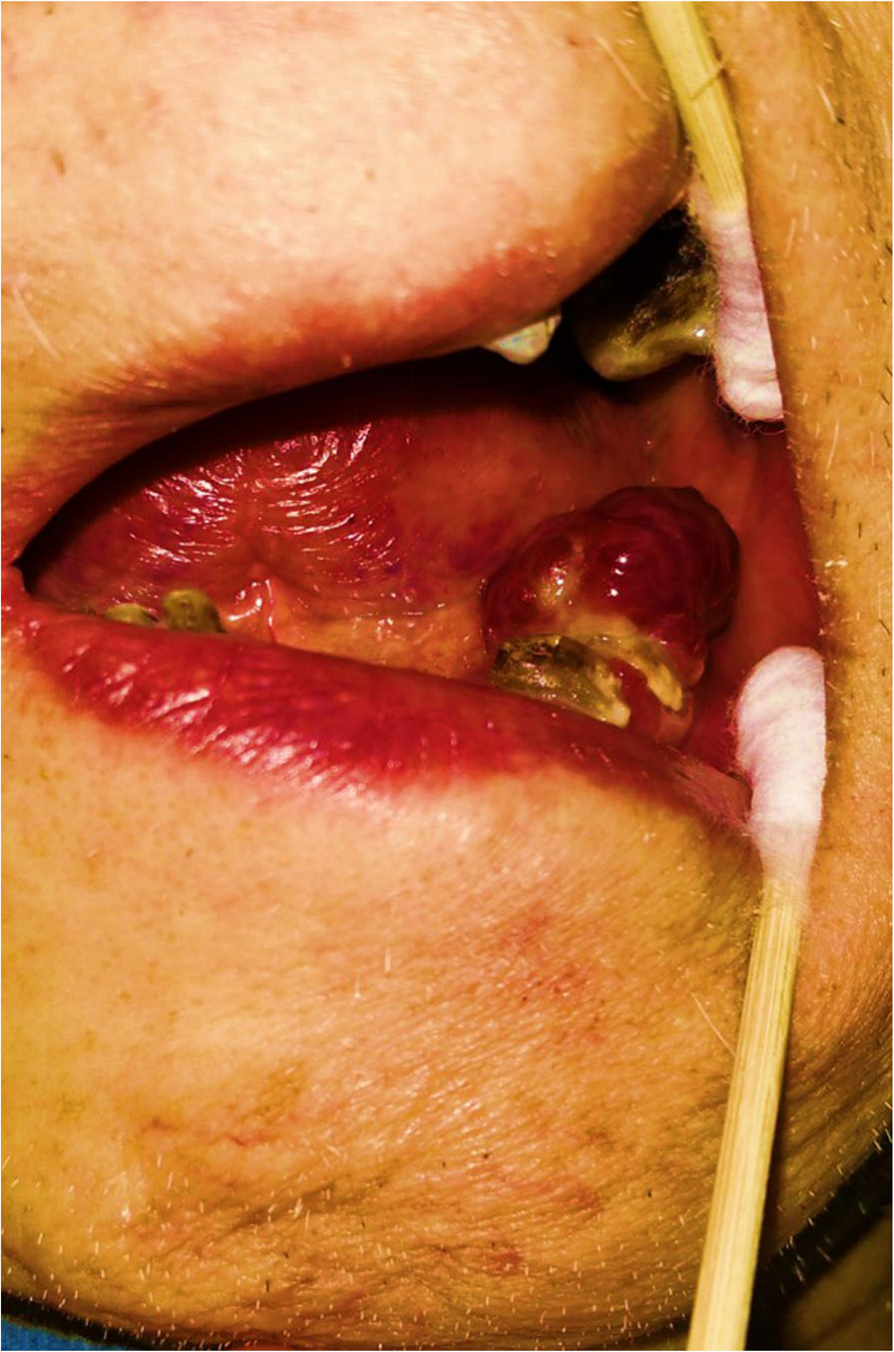
The gingival metastatic tumor image. A reddish, fragile gingival lump, measuring 5.0 × 5.0 × 4.0 cm was found on the left lower gingiva

Oral examinations discovered a reddish soft tissue swelling measuring 2.5 × 2.5 × 2.0 cm with a well-defined border on the gingiva adjacent to the lower left mandible. The mass was bleeding slightly. The mass was provisionally diagnosed as a primary gingival tumor. Considering his poor organ function that prohibited active treatment, such as partial hepatectomy or chemoembolization, the patient decided, with his guardians, to receive palliative treatment for the primary liver cancer. Regarding the treatment for the gingival mass, a stomatologist was consulted; his advice was that the tumor could be resected to relieve any trouble with chewing or eating resulting from the existence of the mass as an oral obstacle. Considering the patient’s poor condition, however, the patient and his guardians decided that he would receive palliative treatment. One episode of profuse bleeding from the root of the gingival lesion occurred and was staunched by local compression. The disease remained relatively stable until considerable progression was observed approximately 1 month after the patient was discharged from the hospital. When the patient was once again admitted to our hospital 2 months later, the mass size had rapidly doubled to 5 × 5 × 4 cm (Fig. 2). Obstructed by the lump, the patient was only able to receive a fluid diet. Unfortunately, with progressed unconsciousness from the sequelae of cerebral infarction, the patient bit the mass open by chance, and profuse bleeding occurred at the residual lesion. Despite pressing continuously to staunch the bleeding and transfusing blood to improve subsequent anemia, the patient’s condition worsened, and he eventually died of multiple organ failure 2 days later.

**Fig. 3:**
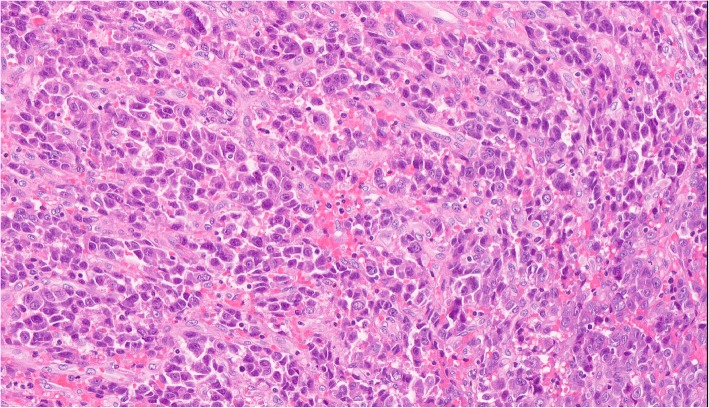
Histopathological staining findings. H&E staining showing oral squamous mucosa with a submucosal proliferation of malignant epithelioid cells arranged in a trabecular architecture. The tumor cells resembled hepatocytes with moderate nuclear atypia and abundant cytoplasm. (H&E; Magnification × 160)

**Fig. 4:**
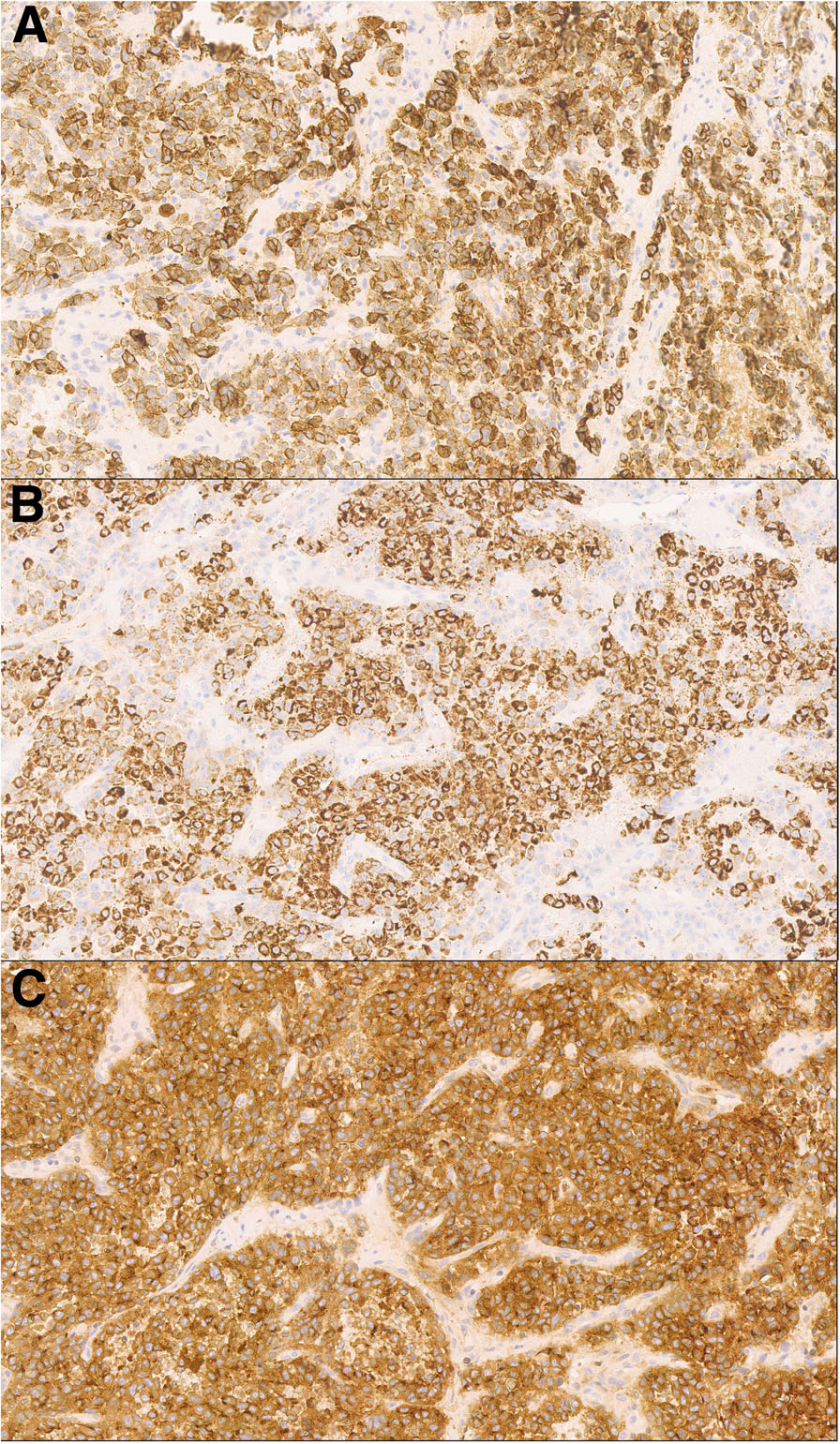
Immunohistochemical findings. Immunohistochemical examinations demonstrated a strong positive reaction to antibodies directed against (**a**) Hepatocyte, (**b**) CAM5.2, (**c**) CD10. (Magnification × 160)

A tissue biopsy from the gingival mass was performed. Histologic examination revealed a squamous epithelium-coated neoplasm dotted with cells that had grown in an invasive trabecular pattern surrounded by a sinusoid network. Largely resembling hepatocytes, the tumor cells with abundant cytoplasm displayed moderate nuclear atypia with some nuclei discernible (Fig. 3). This microscopic appearance was compatible with the diagnosis of HCC. Immunohistochemistry (IHC) tests demonstrated that the tissue showed strong positive reactions to antibodies against hepatocytes (Fig. 4a), CAM5.2 (Fig. 4b), and CD10 (Fig. 4c) and low affinity to antibodies against glypican-3, arginase-1, thyroid transcription factor-1, and cytokeratin-7. Ultimately, the gingival mass was definitively diagnosed as a metastasis from HCC.

## Literature review

### Literature

Any searchable literature in the PubMed, Web of Science and Google Scholar databases concerning gingival metastasis from HCC, whatever language it was published in, is included. The search term used was “cancer” OR “carcinoma” OR “tumor” OR “neoplas*”) AND (“liver” OR “hepatic” OR “hepatocellular”) AND “metasta*” AND “gingiv*”. The references attached to all searched articles serve as a secondary source. A total of 30 cases, including the present case, were reported from 1964 to 2019 and were collected for analysis, including 26 English and four non-English case reports. Twenty-two cases were reported in the twenty-first century. Available data regarding clinical and pathological characteristics are summarized in Tables 1 and 2.

**Table 1:** Cases of gingival metastasis by hepatocellular carcinoma with survival data in reviewed literatures

Case Number	Source	Gender	Age	Gingival neoplasm as first sign	Metastasis besides gingiva	Lesion location on gingiva	Major lesion manifestation	Preexisting Hepatopathy	Differentiation Grade	Primary tumor therapy	Giginval tumor therapy	Truncated/Overall survival
1	Lapeyrolerie, 1964	Male	56	Yes	Lungs, Pancreas, Adrenal, LNs	Upper	Ulceration	ND	Poor	ND	ND	2/2 months
2	Radden, 1966	Male	51	Yes	Lung, LNs, Skin, Peritoneum	Left Upper	Swelling	Alcoholic cirrhosis	Undifferentiation	None	Resection	2/2 months
3	Lund, 1970	Male	52	No	Lungs	Lower	ND	ND	High	Hepatectomy	Resection	39/15 months
4	Kuga, 1976	Male	65	No	None	Left Upper	Bleeding	None	Poor	None	Resection	1/1 month
5	Wedgwood, 1979	Male	56	No	None	Right Upper	Bleeding	Alcoholic cirrhosis	Moderate	None	Resection	6/1 months
6	Morishita, 1984	Male	64	No	Lungs, Adrenals, LNs	Central Upper	Bleeding	Liver cirrhosis	Moderate	TACE	Resection	22/1 months
7	Kanazawa, 1989	Female	78	Yes	Skull, Lumbar vertebrae	Right Upper	Bleeding	Post-hepatitis cirrhosis	Moderate	None	Resection	4/4 months
8	Llanes, 1996	Male	66	Yes	None	Lower	Ulceration, Bleeding	None	Moderate	None	Resection	5/5 months
9	English, 2000	Male	44	No	Nasal cavity, Bone	Left Upper	Swelling	Post hepatitis C cirrhosis, Liver transplant	ND	Chemotherapy	Chemotherapy	> 24/> 3 months
10	Maiorano, 2000	Male	70	Yes	Lungs, Brain	Lower	Slow enlarging	None	Moderate	TACE	Resection	8/8 months
11	Papa, 2001	Male	55	No	Manaible anailiac bones, Ribs, Scapule, Pleura, Brain	Lower	Uncontrolled Bleeding	Post hepatitis C cirrhosis	Moderate	Liver tumor alcoholization, TACE	Segmental resection of left mandible	> 92/> 8 months
12	Choi, 2002	Male	59	No	Lungs	Left Upper	Ulceration, Bleeding	None	Moderate	Partial hepatectomy, TACE	Resection	2/2 months
13	Ramirez, 2003	Male	65	No	None	Central Upper	Bleeding	Alcoholic cirrhosis	Moderate	Chemotherapy	Resection	15/8 months
14	Rim, 2003	Female	70	No	None	Central Lower	Easy bleeding	Post hepatitis B cirrhosis	Moderate	TAE	Resection	> 7/> 7 months
15	Pires, 2004	Male	60	No	Lung, Skin of multiple sites	Central-Left Lower	Ulceration	Post hepatitis B cirrhosis	Moderate	Partial hepatectomy, TACE	Partial resection	38/6 months
16	Arai, 2004	Female	72	No	Cardiac muscle, Abdominal LN	Central Upper	Rapid enlarging, Bleeding	Post hepatitis C cirrhosis	Moderate	TACE	Radiotherapy	10/1 months
17	Elkhoury, 2004	Female	44	Yes	Left cervical LN, Small bowel, Submental	Right and Left lower	Occasional bleeding	ND	Undifferentiation	Palliative care	Palliative care	5/5 months

*ND* not described, *LN* lymph node, *TACE* transarterial chemoembolization, *TAE* transcatheter arterial embolization.

^∗^Truncated survival, the period from onset of gingival metastasis to death

**Table 2:** Cases of gingival metastasis by hepatocellular carcinoma with survival data in reviewed literatures

Case Number	Reference	Gender	Age	Gingival neoplasm as first sign	Metastasis besides gingiva	Lesion location on gingiva	Major lesion manifestation	Preexisting Hepatopathy	Differentiation Grade	Primary tumor therapy	Giginval tumor therapy	Truncated/Overall survival
18	Kuo, 2006	Male	57	Yes	Brain, Lung	Right Upper	Rapid enlarging	Post hepatitis B cirrhosis	Moderate	None	Resection	11/1 months
19	Rai, 2009	Male	82	Yes	None	Left Lower	Rapid enlarging	None	Poor	ND	ND	ND
20	Huang, 2009	Male	60	Yes	Right anterior chest wall	Right upper	Rapid enlarging	ND	Moderate	ND	ND	ND
21	Inaba, 2011	Male	80	No	Lungs	Right Lower	Uncontrolled Bleeding	Post hepatitis C cirrhosis	Poor	TAE	TAE	92/1 months
22	Poojary, 2011	Male	70	Yes	Lungs, Adrenal glands, Thoracic vertebra	Right Lower	Rapid enlarging	ND	Moderate	Palliative treatment	Palliative treatment	ND
23	Terada, 2011	Male	55	Yes	None	Upper Left	Bleeding	Post hepatitis C cirrhosis	High	Chemotherapy	Chemotherapy	ND
24	Greenstein, 2013	Male	68	No	Lungs	Central Upper	Continuous bleeding	Post hepatitis B cirrhosis	Moderate	Chemotherapy	Resection	ND
25	Gentile, 2013	Male	80	No	Lungs, Penis	Right Lower	Bleeding	ND	Moderate	Partial hepatectomy, Radiofrequency ablation, Chemotherapy	None	26/3 months
26	Chen, 2014	Male	58	No	Lungs, Renal	Right Upper to Left lateral	Rapid enlarging, Occasional bleeding	ND	Moderate	Partial hepatectomy, Chemotherapy	Chemotherapy, Radiotherapy	ND
27	Gong, 2015	Male	43	No	ND	Right Upper	ND	ND	Moderate	None	Resection	> 24/> 12 months
28	Kwon, 2016	Male	50	Yes	ND	Left Upper	Rapid enlarging, Bleeding, Pain	Post hepatitis B cirrhosis	Undifferentiation	None	None	2/1 weeks
29	Xue, 2017	Male	60	No	Lungs, Brain	Right Upper	Pain	Post hepatitis B cirrhosis	Poor	Chemotherapy, Sorafenib, TACE	Resection	13/2 months
30	Present case	Male	87	No	Lungs	Left Upper	Bleeding	Post hepatitis B cirrhosis	Moderate	None	None	3/2 months

*ND* not described, *LN* lymph node, *TACE* transarterial chemoembolization, *TAE* transcatheter arterial embolization.

^∗^Truncated survival, the period from onset of gingival metastasis to death

### Age and sex

The disease occurred among people between the ages of 43 and 87, with a median age of 60. Most cases were male with a male-to-female ratio greater than 6:1 (26:4) (Table 3).

**Table 3:** Demographics and characteristics of gingival metastases from hepatocellular carcinoma cases reported between 1964 and 2018

Background data	Total cases (*n* = 30)
Age, years, median (range)	60 (43–87)
Male, gender, n (%)	26 (86.7)
Gingival Lesion as first sign, n (%)	12 (40.0)
Metastatis sites, n (%)	
Gingiva	30 (100.0)
Lungs	15 (50.0)
Lymph nodes	5 (16.7)
Brain	4 (13.3)
Adrenals	3 (10.0)
Skin	2 (6.7)
Vertebrae	2 (6.7)
Kidney	1 (3.3)
Penis	1 (3.3)
Small bowel	1 (3.3)
Major Gingival Manifestation, n (%)	
Bleeding	17 (56.7)
Rapid enlarging	7 (23.3)
Ulceration	4 (13.3)
Swelling	2 (6.7)
Pre-existing Hepathology, n (%)	
Post hepatitis B cirrhosis	7 (23.3)
Post hepatitis C cirrhosis	5 (16.7)
Alcoholic cirrhosis	3 (10.0)
Transfusion hepatitis cirrhosis	1 (3.3)
None	5 (16.7)
ND^a^	9 (30.0)
Differention Grade^b^, n (%)	
Moderate	19 (63.3)
Poor	5 (16.7)
Undifferentiation	3 (10.0)
High	2 (6.7)
ND^a^	1 (3.3)
Gingival lesion location, n (%)	
Upper	19 (63.3)
Lower	11 (36.7)
Left	11 (36.7)
Central	6 (20.0)
Right	11 (36.7)
ND^a^	2 (6.7)

^a^ND, not described. ^b^Differention Grade, evaluated according to World Health Organization Classification of Tumours by International Agency for Research on Cancer

### Preexisting hepatopathy

Twelve cases had a history of posthepatic cirrhosis; seven developed from chronic hepatitis B infection and five developed from chronic hepatitis C infection. In addition, three cases were diagnosed with alcoholic cirrhosis, and one case was diagnosed with transfusion hepatitis cirrhosis. For the remaining cases, five were reportedly free of hepatopathy, and nine lacked a description of a previous history of liver disease (Table 3).

### Gingival metastatic site manifestation

Twelve (40.0%) cases presented with no primary HCC symptoms; their first manifestation was gingival lesions. The distributions of the metastatic lesions on the gingivae are summarized in Table 3. Regarding the location on the gingiva, the lesion presented with a preference for the upper (19, 63.3%) compared to the lower gingiva (11, 36.7%) but no preference for the left, central, or right gingiva. Bleeding and rapid growth were the most common manifestations (Table 3).

### Pathological differentiation grade

The tumor differentiation grade was evaluated in compliance with the World Health Organization Classification of Tumors by the International Agency for Research on Cancer. One case was excluded due to its lack of description. Among the remaining 29 cases, 19 (63.3%), 5 (16.7%), 2 (6.7%), and 3 (10.0%) cases were assessed as moderate, poor, high differentiation, and undifferentiated, respectively. (Table 3).

### Metastasis to sites other than the gingiva

In addition to the gingiva, the most frequent metastatic site was the lungs, followed by the lymph nodes, brain, adrenal glands and others, in descending order by frequency (Table 3).

### Survival analysis

**Fig. 5:**
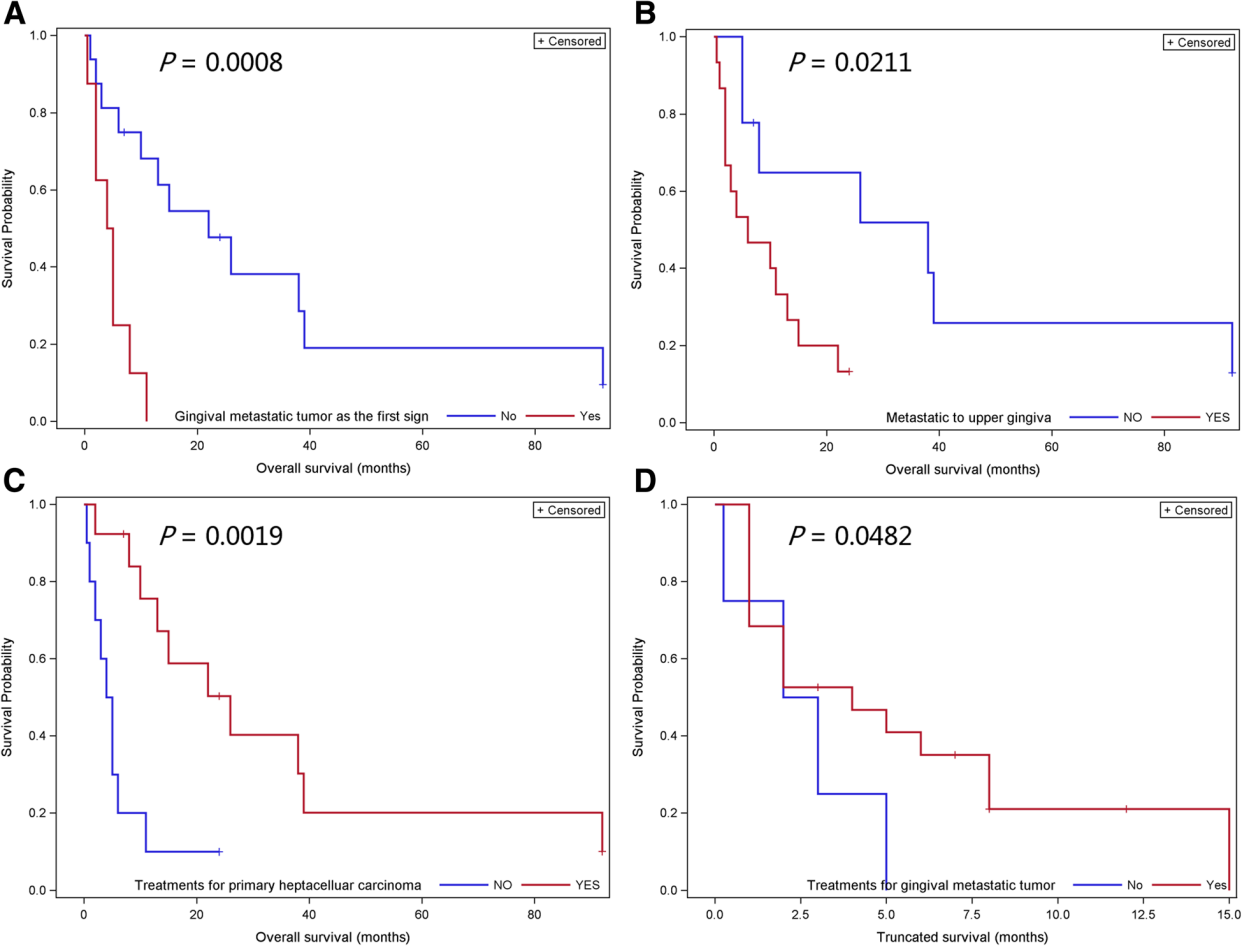
Kaplane-Meier curves for primary hepatocellular carcinoma with metastasis to the gingiva. The curves illustrate (**a**) the overall survival according to gingival metastatic tumor as the first sign, (**b**) the overall survival according to metastasis to upper gingiva, (**c**) the overall survival according to treatments for primary hepatocellular carcinoma, and (**d**) the truncated survival according to treatments for gingival metastatic tumor

Data regarding overall survival and truncated survival were analyzed. Overall or truncated survival was defined as the period from the onset of HCC or gingival metastasis to death, respectively. Six cases with incomplete data were discarded. The remaining twenty-four cases were included in the survival analysis using SAS software (SAS v9.4; SAS Institute, NC, USA). Survival analysis indicated that gingival lesions as the first sign of HCC (*P* = 0.0008, Fig. 5a) and located on the upper gingiva (*P* = 0.0211, Fig. 5b) presented worse overall survival. Treating the primary HCC improved overall survival (*P* = 0.0019, Fig. 5c), while treating the metastatic gingival tumor improved truncated survival (*P* = 0.0482, Fig. 5d).

## Discussion and conclusions

According to a large-scale global investigation of cancers [1], hepatocellular carcinoma (HCC) ranked sixth in cancer incidence and fourth in cancer mortality worldwide. Despite significant mortality reductions in East Asian countries, such as China, Korea, and Japan, HCC remains the third most common and fatal cancer. Over 50% of HCC patients had extrahepatic metastases, most frequently affecting the lungs, skeleton, brain, abdominal lymph nodes [34]. Metastasis of HCC to the gingiva was believed to be extremely uncommon. However, the rarity of gingival metastasis may be overestimated; some cases published in either English [3, 17–33] or non-English [21, 31] journals were not covered by the major literature databases. Some cases may not be reported at all due to potential misdiagnosis. Some cases first manifested as only gingival lesions [21, 24, 25, 27, 28, 33] or mimicked benign gingival disease [14, 22], both of which would lead to misdiagnosis, especially in the absence of a biopsy and pathological examination.

Gingival metastasis can originate from a wide range of primary sites, including lung, breast, kidney, bone, colorectal, adrenal, and liver [35]. The possible pathophysiological mechanism of HCC metastasis to the gingiva remains to be elucidated. The hematogenous route by invasion of the hepatic arterial or portal venous branches is believed to be the preferred mode for oral metastasis [36–38], although, in some cases, metastatic pulmonary tumors are absent [1, 7, 9–11, 13–15, 19, 20, 22, 23, 28, 32, 33]. Among those cases, the valveless vertebral venous plexus (Batson’s plexus) has been proposed as a mechanism for bypassing filtration through the pulmonary, inferior caval and portal venous circulations [39, 40]. This pathway may be the most likely pathway responsible for HCC metastasis to the gingiva without pulmonary metastasis. In addition to the Batson’s plexus, the other possible routes of gingival metastasis include arterial, venous, and lymphatic circulations [6]. In light of the fact that liver cirrhosis presents in over 50% of HCC patients with metastatic gingival tumors, we cautiously propose a hypothesis that the altered hemodynamics subsequent to esophageal varices may be one of potential pathways for oral metastases, particularly in HCC patients with liver cirrhosis with incomplete compensation.

So far, at least 30 cases of gingival metastasis from HCC have been retrieved from the existing literature sources. Analyzing these cases can help us gain new insights into the clinical and pathological characteristics of gingival metastasis in HCC. First, our present analysis demonstrates a remarkable sex preference in the occurrence of gingival metastasis from HCC. The ratio of male to female is greater than 6:1 (26/4), which far outweighs the overall male-to-female ratio of approximately 3:1 in liver cancer incidence [1]. These inconsistencies raise questions as to whether the relatively poorer general health habits or oral health behaviors among males, such as smoking and drinking, as revealed in a study [41], may favor the pathogenesis of gingival metastasis from HCC. Pathogenesis of this special metastasis is thought to be associated with oral inflammation, such as gingivitis, that possibly attracts migration and adhesion of cancer cells to the gingiva [38]. Chronic inflammation has been involved in various steps of tumorigenesis, including cellular transformation, survival, proliferation, invasion, angiogenesis, and metastasis [42, 43]. The rich capillary network of the chronically inflamed gingiva and the presence of some inflammatory molecules may favor the progression of metastatic cells [38]. Future investigation of this possible mechanism remains to be conducted.

Moreover, according to our survival analysis, patients with a gingival mass as the first sign of HCC had extremely poor survival. The concurrent multiple extrahepatic metastases may have contributed to this poor survival observation. However, among those HCC cases with gingival lesions as the first sign, distant metastasis outside the gingiva was not reported in three cases [10, 24, 28]. In this scenario, the delayed diagnosis and treatment, to some extent resulting from the absence of indications of underlying liver cancer, may worsen survival. This further raises the importance of early diagnosis and treatment of a potential gingival metastasis from HCC or other distant tumors. A timely biopsy is necessary for any neoplasm, even if it resembles a benign lesion [9, 14].

In addition, HCC is more likely to spread to the upper gingiva than the lower gingiva. Looking into the anatomy, we find several structural factors for this distribution preference. The anatomical characteristics of the arteries supplying blood to the gingivae may contribute to the difference. The upper gingiva accepts blood through two main arteries, namely, the superior dental artery and the infraorbital artery. The two arteries, as direct extensions of their stem artery (maxillary artery), have wider diameters and larger blood volumes [44]. Meanwhile, the lower gingiva only accepts blood through one smaller artery called the inferior dental artery, which is a thinner branch of the stem artery. The increase in blood flow may increase the risk of implantation by circulating tumor cells for the upper gingiva.

Early diagnosis of a metastasized gingival mass from underlying primary cancer was critical to the patients’ prognosis. However, misdiagnosis or a missed diagnosis could arise from several factors. First, the low incidence rate and indistinctive manifestation (bleeding, swelling, ulceration, etc.) posed fresh challenges to physicians in acknowledging this rare disease. Second, the deceiving characteristics of the gingival lesions, for instance, mimicking a pyogenic granuloma [14, 22], would make physicians overlook the necessity of a biopsy. However, a gingival mass’s characteristic of rapid growth can put physicians on high alert for a malignancy. As reported in the present case, the gingival lesion was first diagnosed as a primary gingival tumor until the biopsy and the pathological test were completed; then, a metastasis from HCC was finally identified.

The main treatments of primary hepatocellular carcinoma involved hepatectomy, chemotherapy, transarterial chemoembolization (TACE), transcatheter arterial embolization (TAE), novel targeted therapy (sorafenib), and combination therapy. The major treatments for the gingiva lesions included resection, chemotherapy, radiotherapy, and TAE. Survival analysis demonstrated that patients receiving treatments for primary cancer or metastatic gingival lesions appeared to have better overall survival or truncated survival. However, there may be biases between the treated and untreated patient groups. For example, about 20% of the previous case reports lack survival information, and the untreated population may have had a poorer performance status, like our present case. The treatment effectiveness for survival remains to be confirmed based on large sample randomized controlled studies.

As an integral part of evidence-based medicine, case reports and literature reviews have profoundly influenced the medical literature, and they continue to advance our knowledge of diseases and help generate hypotheses to conduct clinical studies and basic research. Despite the relatively small sample size, this case report and literature review may be valuable for physicians to update their knowledge for their daily practice. Enhanced recognition, early diagnosis, and appropriate management of gingival metastasis may help improve the overall outcomes for this distinct subgroup of HCC. Further retrospective or prospective studies with a larger sample size of patients are still required.

Gingival metastasis from primary liver cancer is rare, and the diagnosis of a gingival metastatic lesion is challenging to clinicians. To avoid potential misdiagnosis, a biopsy is mandatory, even if no distinct clinical presentation is observed. Early diagnosis and treatments for primary liver cancer or metastatic gingival lesion may improve survival expectations.

## Data Availability

The datasets used during the current study are available from the corresponding author on reasonable request.

## Abbreviations

AFP: Alpha-fetoprotein
APTT: Activated partial thromboplastin time
CA-125: Carbohydrate antigen-125
CT: Computed tomography
GGT: Gamma-glutamyl transpeptidase
HCC: Hepatocellular carcinoma
IHC: Immunohistochemistry
INR: International normalized ratio
MRI: Magnetic resonance imaging
TACE: Transarterial chemoembolization
TAE: Transcatheter arterial embolization
TBIL: Total bilirubin
